# High-resolution mapping of an aortic sinus cusp for two outflow tract premature ventricular contractions

**DOI:** 10.1016/j.jccase.2025.06.002

**Published:** 2025-06-21

**Authors:** Yuto Tamura, Kentaro Ozu, Takafumi Oka, Takayuki Sekihara, Tomohito Ohtani, Yasushi Sakata

**Affiliations:** a Department of Cardiovascular Medicine, Osaka University Graduate School of Medicine, Suita, Osaka, Japan; b Department of Cardiovascular Medicine, Higashiosaka Medical Center, Higashiosaka, Osaka, Japan

**Keywords:** Aortic sinus cusp, Premature ventricular contraction, Catheter ablation, High-resolution mapping, Case report

## Abstract

In catheter ablation for outflow tract premature ventricular contraction (PVC), electrical mapping of the aortic sinus cusp (ASC) provides critical information for identifying the origin of the PVC. The OCTARAY™ (Biosense Webster, Diamond Bar, CA, USA), a multispline 48-polar electrode catheter with TRUEref™ technology, fits the shape of the ASC and enables detailed and prompt high-resolution ASC mapping without inducing PVCs due to mechanical stimulation. We present a case of successful catheter ablation for two similar PVCs from the ASC based on activation and voltage mapping using TRUEref™ technology. High-resolution mapping could be helpful for the visual assessment of the detailed wavefront propagations of PVC.

**Learning objective:**

In catheter ablation for outflow tract premature ventricular contraction (PVC), mapping the aortic sinus cusp is useful to identify the origin; however, it is sometimes challenging with a conventional electrode catheter. High-resolution mapping using OCTARAY™ might be helpful for detailed assessment of outflow tract PVC.

## Introduction

In catheter ablation for outflow tract (OT) premature ventricular contraction (PVC) and left ventricular (LV) summit PVC, electrical mapping of the aortic sinus cusp (ASC) provides critical information for identifying the PVC origin [[1], [2], [3]]. The OCTARAY™ (Biosense Webster, Diamond Bar, CA, USA), a multispline 48-polar electrode catheter, enables prompt high-resolution electrical mapping [4]. This case study aimed to assess the detailed wavefront propagations of two PVCs with similar morphologies using high-density mapping with OCTARAY™ within the ASC.

## Case report

**Fig. 1:**
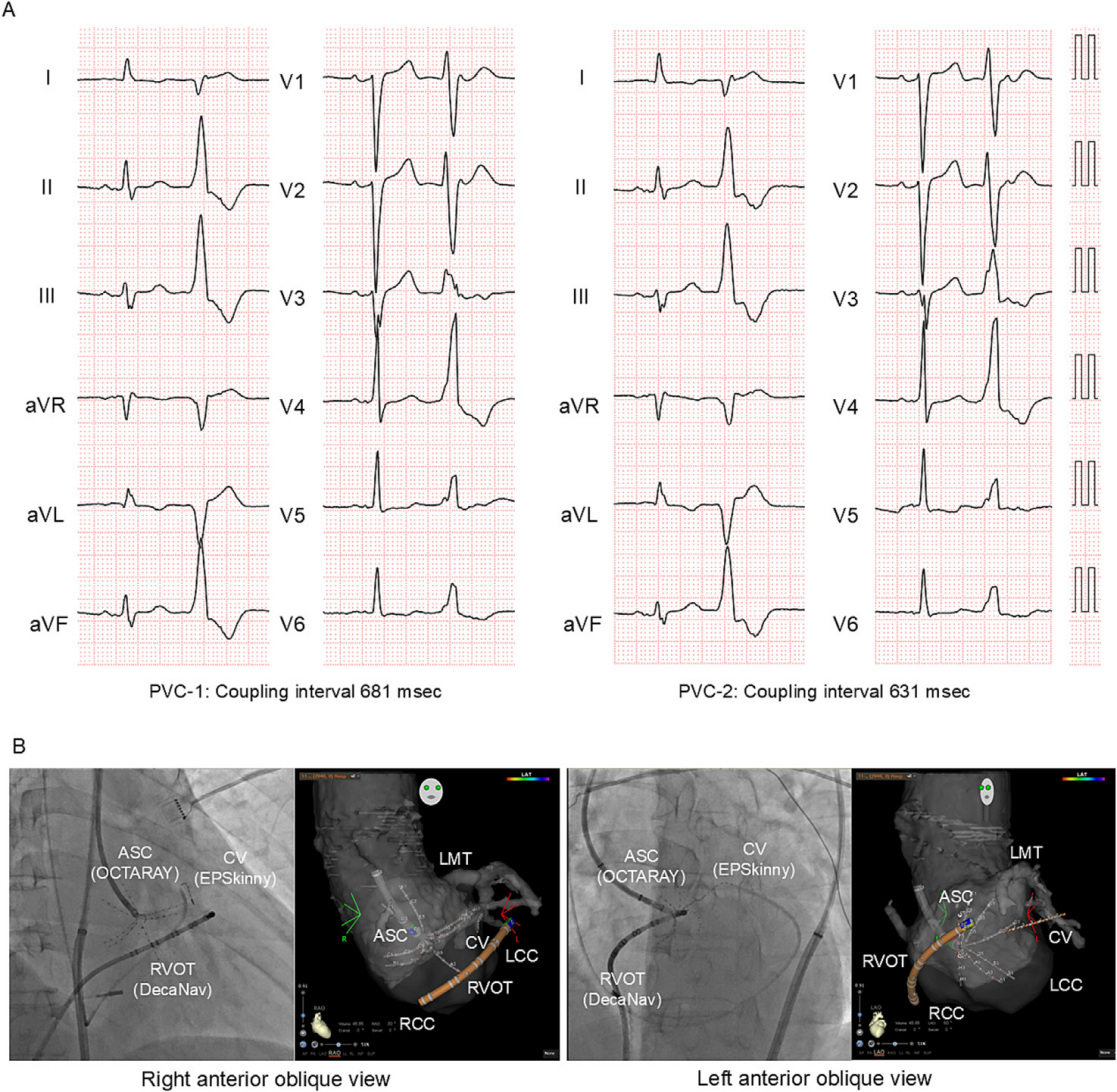
Electrocardiogram and catheter position. (A) Twelve-lead electrocardiogram of PVC-1 and PVC-2. (B) Catheter position during aortic sinus cusp mapping using DecaNav™, EPSkinny™, and OCTARAY™. LMT, left main trunk; ASC, aortic sinus cusp; LCC, left coronary cusp; RCC, right coronary cusp; RVOT, right ventricular outflow tract; CV, communicating vein; PVC, premature ventricular contraction.

**Fig. 2:**
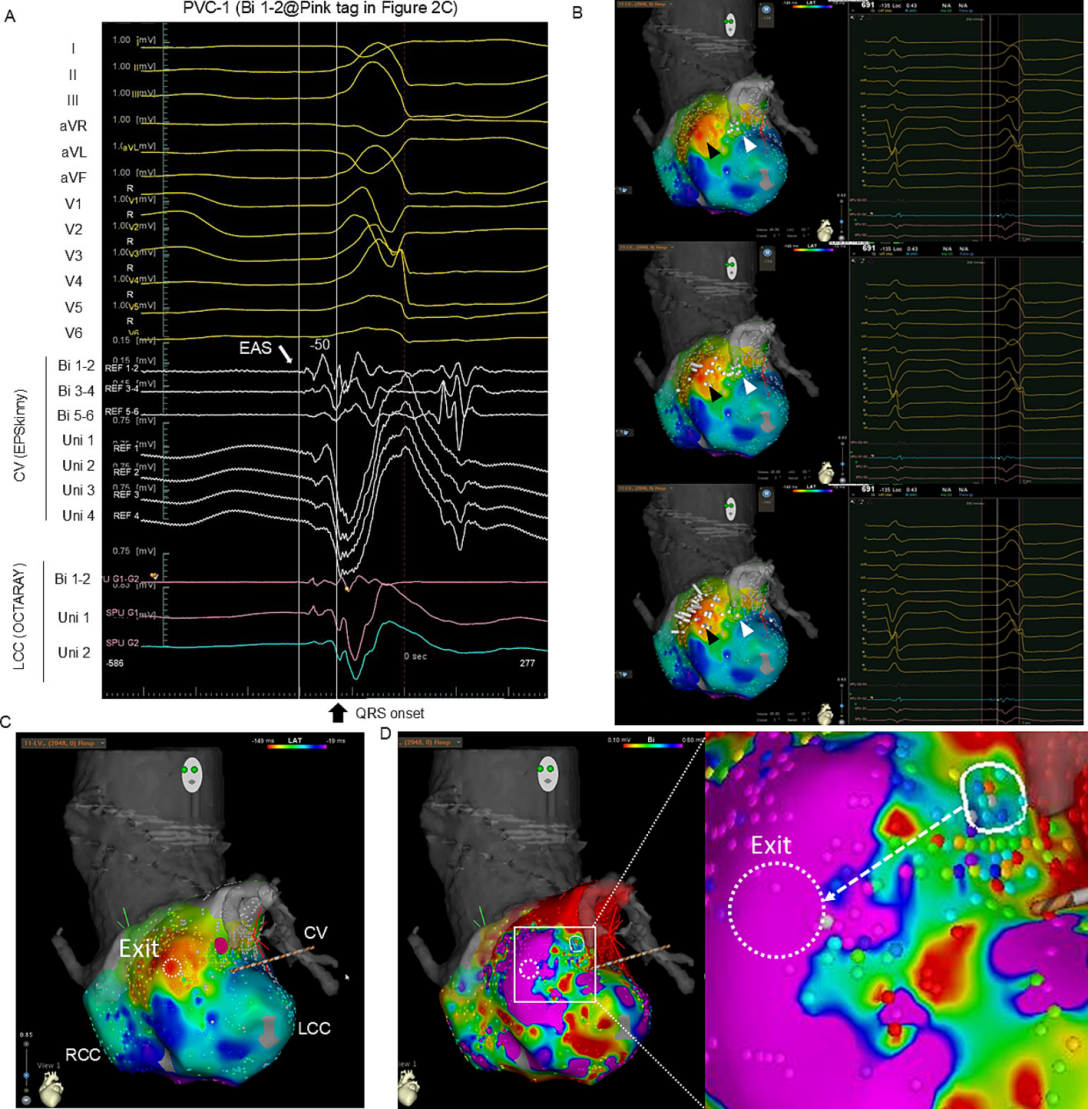
Premature ventricular contraction-1. (A) Electrocardiogram of PVC-1. The LCC Bipolar 1–2 was positioned at the pink tag in (C). (B) Ripple mapping of PVC-1. Upper: pre-potential was recorded at the white arrowhead, auto-annotated early activation site was recorded at the black arrowhead, mid: propagation through the preferential pathway to endocardial exit (black arrowhead), lower: centrifugal activation from endocardial exit. (C) PVC-1 activation mapping. The pre-potential was recorded on the pink tag. (D) Bipolar voltage mapping of PVC-1. The white dashed circle shows the endocardial exit. The white dotted arrow shows the assumed preferential pathway from PVC-1 origin to the exit. PVC, premature ventricular contraction; EAS, earliest activation site; CV, communicating vein: RCC, right coronary cusp; LCC, left coronary cusp.

**Fig. 3:**
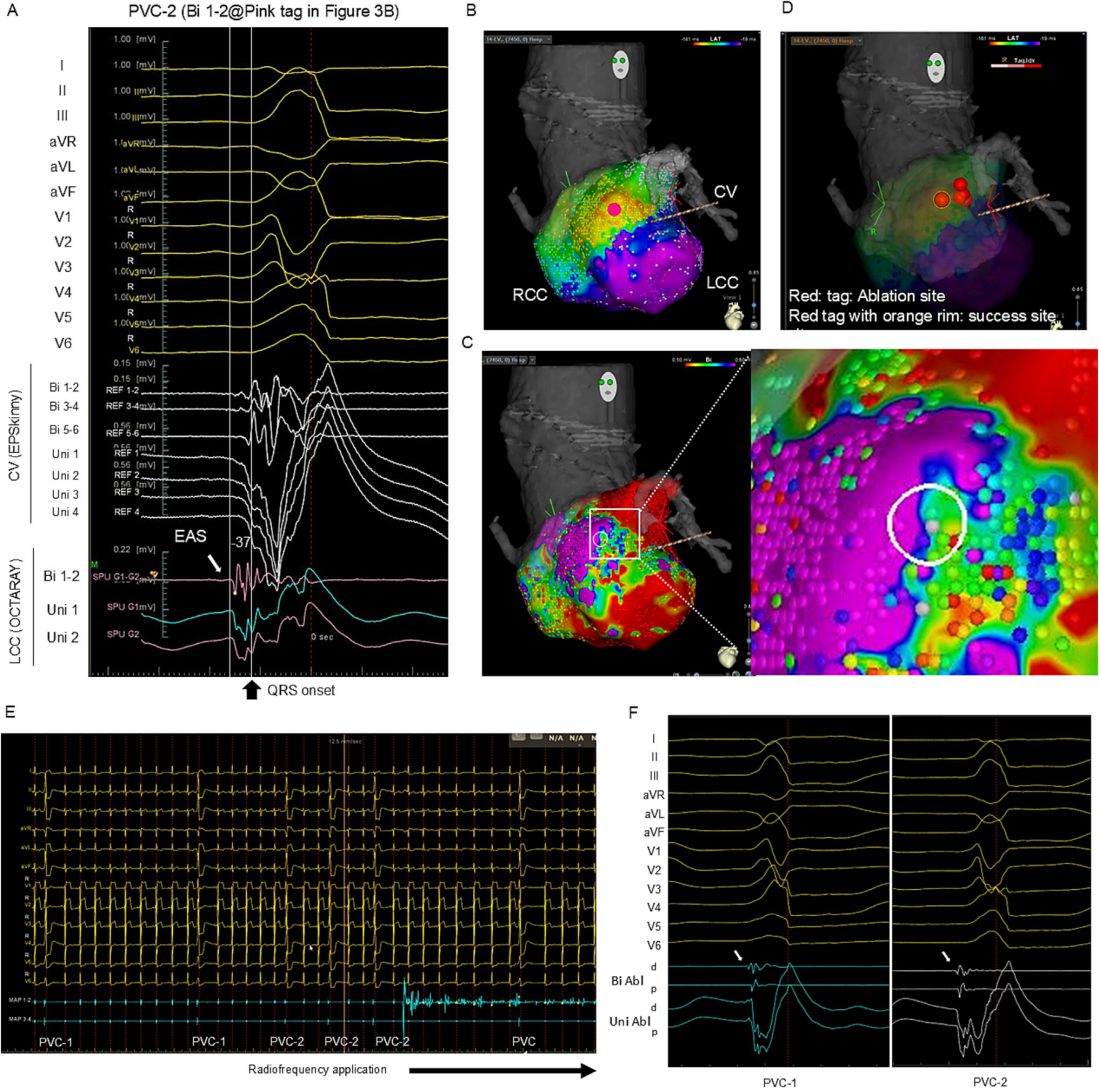
Premature ventricular contraction-2. (A) Electrocardiogram of PVC-2. The LCC Bipolar 1–2 was positioned at the pink tag in (B). (B) PVC-2 activation mapping. (C) PVC-2 voltage mapping. The white circle shows the earliest activation site of PVC-2. (D) Ablation site. The red circle with an orange rim shows the success site. (E) Twelve-lead electrocardiogram just before and after radiofrequency application. (F) The intracardiac electrocardiogram at the success site. Left: PVC-1; right: PVC-2. PVC, premature ventricular contraction; CV, communicating vein: RCC, right coronary cusp; LCC, left coronary cusp; EAS, earliest activation site.

A 65-year-old man with a history of congestive heart failure with reduced left ventricular ejection fraction (LVEF: 23 %), and persistent atrial fibrillation (AF) was referred to our hospital. After AF ablation, LVEF improved to 40 %, but PVCs (burden: 25 %) appeared. Two types of PVC were observed (Fig. 1A); both exhibited a left bundle-branch block morphology with an inferior axis and precordial transition in V3. We performed catheter ablation for the PVCs. CARTO™ 3 System Version 7 (Biosense Webster) was used. A decapolar catheter (DecaNav, Biosense Webster) was positioned in the right ventricular outflow tract (RVOT), and a 1.6-Fr hexapolar electrode catheter (EPSkinny, Kaneka, Tokyo, Japan) was placed in the communicating vein (CV). Because the potentials in the RVOT were delayed compared to those in the CV for both PVCs, we positioned an OCTARAY™ (inter-electrode spacing: 3-3-3-3-3) on the left coronary cusp (LCC), which was located opposite the CV (Fig. 1B). The earliest activation site (EAS) of PVC-1 was at the CV (EPSkinny) with a bipolar potential preceding QRS onset by 50 ms (Fig. 2A). The two PVCs were simultaneously mapped with the CONFIDENSE™ Parallel Mapping module. After 27 min, 2994 points for PVC-1 and 7450 points for PVC-2 were acquired. Mechanical PVCs that interfered with the mapping occurred infrequently (11 PVCs during 27 min). In the bipolar activation map of PVC-1 (Fig. 2B), the auto-annotated EAS (black arrowhead) was located at the anterolateral LCC. In ripple mapping (showing bars above 0.01 mV; Video 1), the earliest excitation (Fig. 2B, white arrowhead) was observed at 10 mm far from the auto-annotated EAS (black arrowhead). The excitation was propagated toward the auto-annotated EAS (Fig. 2B, middle) and spread centrifugally (Fig. 2B, lower). At the earliest ripple signal site (ERS) (Fig. 2C, pink tag), a bipolar pre-potential with a positive unipolar deflection was observed (Fig. 2A, LCC Bi 1–2), which was at the opposite end of the CV (Fig. 2A, CV Bi 1–2). In the bipolar voltage mapping [scar ≤0.1 mV, low voltage area (LVA) ≤0.5 mV] (Fig. 2D), the auto-annotated EAS (white dashed circle) was amid the normal voltage area (NVA), while the bipolar pre-potential recorded site (white circle) was amid the LVA. Based on these findings, the origin of PVC-1 was considered the deep epicardium near the CV, possessing a preferential pathway toward the endocardial exit in the anterolateral LCC. Subsequently, the origin of PVC-2 was assessed. The local unipolar waveform at the EAS was a QS pattern without a pre-potential (Fig. 3A, LCC Uni 1–2). Ripple mapping showed no preceding excitation (Video 2). Since the EAS was concordant with the ERS, PVC-2 was considered to have a superficial endocardial origin (Fig. 3B). In bipolar voltage mapping of PVC-2 (Fig. 3C), the EAS (white circle) was at the border between LVA and NVA. Therefore, PVC-1 of deep origin and PVC-2 of superficial endocardial origin were considered different. We applied radiofrequency (RF) to PVC-2 of superficial endocardial origin using Thermocool SMARTTOUCH-SF™ (Biosense Webstar) (Fig. 3D, red tag with orange rim). The two PVCs occurred constantly before RF application (Fig. 3E). The ablation catheter recorded potentials preceding QRS onset by 35 ms with a unipolar QS pattern during PVC-2 and preceding QRS onset by 20 ms without a pre-potential during PVC-1 (Fig. 3F). Immediately after the first RF application, PVC-2 was suppressed, whereas PVC-1 disappeared 8 s later. RF application (35 W, contact force >10 g) was continued for up to 60 s. Since the PVCs terminated, additional ablation at the ERS of PVC-1 was not performed. After an additional RF application, ectopy did not recur during a waiting time of 30 min, including the isoproterenol challenge. The successful ablation site was considered close to the deep origin or isolated preferential pathway of PVC-1, resulting in delayed PVC-1 elimination. Six months later, LVEF improved to 45 %. Finally, the patient was diagnosed with arrhythmia-induced cardiomyopathy superimposed on idiopathic dilated cardiomyopathy.

## Discussion

Detailed ASC mapping using conventional electrode catheters is challenging. Although the coronary cusps consist of fibrous tissues and scarce myocardium, the critical far-field signal from adjacent myocardial structures, such as the LVOT and RVOT, is often recorded as a tiny pre-potential electrogram [1,5]. PENTARAY® (Biosense Webster) and INTELLAMAP ORION™ (Boston Scientific, St Paul, MN, USA) have been recently reported as useful in OT-PVC ablation [6,7]. This case study demonstrated the successful ablation of two OT-PVCs from the ASC using high-resolution mapping with OCTARAY™. The TRUEref™ technology of OCTARAY™ using the central isolated shaft electrode as an indifferent electrode enables visualization of sharp and clear local potentials on unipolar electrography with no relevant baseline drift, resulting in a clear bipolar potential [4,7,8]. OCTARAY™ is suitable for ASC mapping for three reasons. First, OCTARAY™, with a soft spline, fitted the ASC's spherical shape. Fast anatomical mapping with OCTARAY™ was almost consistent with the shape of the aortic roots depicted on the computed tomography image. Second, OCTARAY™ rarely induces mechanical PVC during the ASC mapping. In the ventricular chamber mapping, spline-type electrode catheters frequently induce mechanical PVC, but the scarcity of myocardium compared to ventricular chambers nullified the disadvantage of OCTARAY™. Third, the TRUEref™ technology enabled the quick acquisition of multiple tiny potentials from deep origins or preferential pathways. By combining it with a parallel mapping module, two PVCs with similar morphologies were mapped for 27 min. Another benefit is the increased number of mapping points using OCTARAY™ with pattern-matching filters.

PVC-1 and PVC-2 had their EASs in the LCC, suggesting two possibilities: one origin with two exits or separate origins for each PVC. Activation mapping depicted the endocardial breakout site of PVC-1 as the endocardial EAS. Ripple mapping identified the ERS where a tiny pre-potential was recorded on the opposite side of the CV remote from the endocardial EAS, suggesting deep mid-myocardial origin. A positive unipolar local pre-potential at the EAS also suggested that the origin of PVC-1 was not at the endocardial EAS. During PVC-2, the endocardial EAS was concordant with ERS, suggesting the initial endocardial activation was close to the PVC origin and associated with higher odds of single-site PVC suppression and successful ablation [9]. Furthermore, PVC-2 had no preceding pre-potential at the ERSs of PVC-2 and PVC-1, suggesting that PVC-2 had a different origin from that of PVC-1. The potential of the CV at PVC-1 EAS was not preceded in PVC-2.

Bipolar voltage mapping was reviewed to assess the arrhythmogenic substrates of both PVCs. Since the optimal voltage cut-off for ASC is unclear, we explored the optimal cut-off value by manipulating the color range manually (Video 3, Video 4) and used a voltage ≤0.5 mV for the bipolar LVA cut-off. The anterolateral LCC was an NVA, while the bottom of the LCC and the coronary ostium area were LVA or scar. The left ventricular outflow tract (LVOT) myocardium directly contacts the aorta at the base of LCC and RCC. The ASC consists of fibrous tissue and scarce myocardium. Considering the contact of the anterolateral portion of the LCC with the LV ostium and the histological evidence of fibrous tissue in coronary cusps [[1], [2], [3]], our bipolar voltage mapping image seemed reasonable. The endocardial exits of PVC-1 and PVC-2 were located amidst the NVA and on the border zone between NVA and LVA, respectively. The area where pre-potential was recorded in PVC-1 was amidst the LVA. LVA may reflect arrhythmogenic diseased myocardium [10]. Bipolar voltage mapping could help identify PVC origin.

Consequently, we assumed that the two PVCs had different origins. First, we targeted PVC-2, considering the conductivity of RF energy. Immediately after the RF application, PVC-2 was eliminated. PVC-1 was eliminated 8 s after PVC-2 elimination, although the success site was remote from the pre-potential area. Delayed RF energy conduction to the PVC-1 origin or its preferential pathway was considered a plausible mechanism.

ASC high-resolution mapping has limitations concerning safety (including coronary artery injury and contraindication for mechanical valves) and mapping configuration (including ripple mapping threshold and voltage cut-off); however, with refinement, it might become a supportive method for comprehensive mapping to determine the true origin of OT-PVC.

## Conclusions

We report successful PVC ablation from the ASC using high-resolution mapping with OCTARAY™. In combination with other electrode signals high-resolution mapping could help advance further understanding of the PVC origin and detailed wavefront propagations.

The following are the supplementary data related to this article.

Video 1 Ripple mapping of premature ventricular contraction-1. Video 1

Video 2 Ripple mapping of premature ventricular contraction-2. Video 2

Video 3 Titration of bipolar voltage threshold of premature ventricular contraction-1. Video 3

Video 4 Titration of bipolar voltage threshold of premature ventricular contraction-2. Video 4

## Consent statement

Written informed consent was obtained from the patient. This study was approved by an ethics committee.

## Funding

None.

## Declaration of competing interest

None.
